# Advantage of the Highly Restricted Odorant Receptor Expression Pattern in Chemosensory Neurons of *Drosophila*

**DOI:** 10.1371/journal.pone.0066173

**Published:** 2013-06-19

**Authors:** Sana Khalid Tharadra, Adriana Medina, Anandasankar Ray

**Affiliations:** Department of Entomology, University of California, Riverside, California, United States of America; Center for Genomic Regulation, Spain

## Abstract

A fundamental molecular feature of olfactory systems is that individual neurons express only one receptor from a large odorant receptor gene family. While numerous theories have been proposed, the functional significance and evolutionary advantage of generating a sophisticated one-receptor-per neuron expression pattern is not well understood. Using the genetically tractable *Drosophila melanogaster* as a model, we demonstrate that the breakdown of this highly restricted expression pattern of an odorant receptor in neurons leads to a deficit in the ability to exploit new food sources. We show that animals with ectopic co-expression of odorant receptors also have a competitive disadvantage in a complex environment with limiting food sources. At the level of the olfactory system, we find changes in both the behavioral and electrophysiological responses to odorants that are detected by endogenous receptors when an olfactory receptor is broadly misexpressed in chemosensory neurons. Taken together these results indicate that restrictive expression patterns and segregation of odorant receptors to individual neuron classes are important for sensitive odor-detection and appropriate olfactory behaviors.

## Introduction

The molecular and cellular organization of an olfactory system consists of several sensor types each expressing one member from a large odorant receptor family. Each sensory neuron type detects different subsets of odors, and connects in a specific receptor-to-neuron-to-glomerulus manner. Despite vastly different receptor families and mechanisms of receptor regulation this highly restricted receptor expression pattern is highly conserved from invertebrates to vertebrates. This architecture provides a sophisticated spatial map of odor activation in the brain, which is thought to be important for detection and discrimination. While a complete understanding of mechanisms that underlie specification of this sophisticated map of receptor expression is not available, multi-layered systems of genetic [1], [2], [3], [4], [5], [6], [7], [8], [9], [10] and epigenetic mechanisms [11], [12], [13] that maintain the restricted “singular” expression of one-receptor-per-neuron [4], [5], [6], [7], [8], [14], [15], [16] have been uncovered recently. The biological advantage of this highly conserved molecular organization has never been tested.

Genetic manipulation to co-express functional odorant receptor genes is intractable in vertebrates, considering the elaborate mechanisms for negative-feedback regulation [3], [15], [16] that impart “singular” expression even to receptor transgenes. However, co-expression of two or more functional odorant receptors is feasible in the model genetic system *Drosophila melanogaster* where negative-feedback mechanisms are absent [7]. In fact there are a few classes of olfactory neurons that co-express two or more *Or* genes [17], [18], in most instances ones that are closely related recent duplications or are expressed from a bi-cistronic mRNA [7]. Over-expression of functional receptors in the adult fly does not suppress expression of the endogenous receptor [7] and can modify behavioral responses [19].

The *Drosophila* larva provides a powerful model to test olfactory behavior since it has a well-characterized olfactory system consisting of a pair of dome sensilla on either side. Each dome is innervated by the dendrites of 21 olfactory neurons (ORNs) that send stereotypical axonal projections to glomeruli in the larval antennal lobes, where the information is collected by 21 second order projection neurons to take to higher brain centers [16], [20], [21], [22], [23], [24], [25], [26]. Moreover, the *Drosophila* larva has previously been utilized as a model to study olfactory competition in a population assay [27]. Using this assay it was effectively demonstrated that mutant flies lacking the broadly expressed odorant co-receptor subunit, *Orco*, have a significant competitive disadvantage [27].

## Results

Previously, we identified a repressive *cis-*regulatory sequence upstream of *Or42a* that refined expression to only one neuronal class in the olfactory system by repressing expression in several other neuron classes in the olfactory and gustatory system [6]. This repressive palindromic sequence is largely conserved across the 12 sequenced *Drosophila* species hinting at some functional advantage to repressing *Or42a* expression in the non-endogenous neuronal classes.

**Figure 1:**
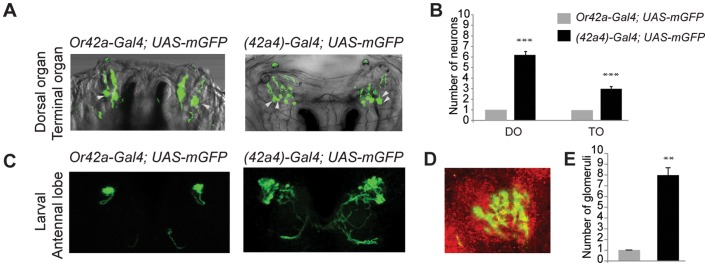
A mutated ***Or42a*** **promoter can drive expression in multiple chemosensory neurons in the larvae.** (**A**) Confocal micrograph Z-projections of olfactory neurons with dendrites innervating the Dome sensillum seen as fluorescent dome structure called the Dorsal Organ, and putative gustatory neurons (marked with arrowheads) with dendrites innervating the Terminal Organ in larvae expressing *UAS-mcd8:GFP* under the control of the wild-type *Or42a-Gal4* or mutant *(42a4)-Gal4* [6] (**B**) Mean number of neurons innervating the Dorsal Organ (DO) and Terminal Organ (TO) in the indicated genotypes. N = 7 (Or42a-Gal4) and N = 13 ((42a4)-Gal4), error bars = s.e.m., T-test, **P<0.0001. (**C**) Representative confocal Z-projections from larval brains of indicated genotypes. (**D**) Zoomed in view of a larval antennal lobe from *(42a4)-Gal4; UAS-mcd8:GFP* counterstained with nc82 (red). (**E**) Mean number of glomeruli labeled by *UAS-mcd8:GFP* driven by the indicated promoters. N = 6 for each sample, error bars = s.e.m., T-test, **P<0.01.

A wild-type promoter construct of *Or42a* drives expression of GFP in one pair of ORNs innervating the larval dome sensillum (Dorsal Organ) and one pair of neurons innervating the Terminal Organ that is associated with tastants (Figure 1A, Video S1) [28]. A promoter construct for *Or42a* with a mutation in the motif de-represses expression in ∼6 pairs of ORNs and ∼3 pairs of gustatory neurons (GRNs) in the larval olfactory system [6] (Figure 1A, Video S2). The number of glomerulus-like structures innervated by neurons labeled by the mutant *(42a4)*-promoter indicates misexpression occurs in ∼7 of the 21 functional ORN classes (Figure 1A,B,C, Video S3, Video S4). These larvae provide an ideal system for analyzing the functional advantage of restricting *Or42a* to one-olfactory receptor-per-neuron since they can be tested in a well-developed olfactory competitive-survival assay [27].

**Figure 2:**
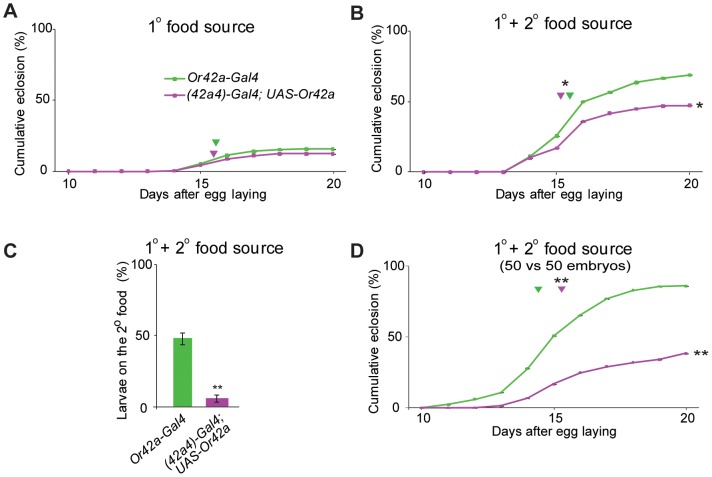
Larvae co-expressing *Or42a* in multiple neurons show reduced survival and failure to exploit a secondary food source in a competitive environment. Mean cumulative rates of eclosion from 50 embryos each of *(42a4)-Gal4* (green line, control) and *(42a4)-Gal4;UAS-Or42a* (magenta line, co-expressing) plotted for days after egg laying in (**A**) a primary limiting food source, and (**B**) a primary and a secondary limiting food source. (**C**) Percentage of larvae accumulating on the second food source at 5 days after egg laying. (**D**) Mean cumulative rates of eclosion from competition between 50 embryos of the two genotypes when presented in a primary and secondary limiting food source as in (**B**). Arrowhead indicates half-maximal eclosion rate for each genotype, T-test, *P<0.05, **P<0.01, N = 6 trials, error bars = s.e.m.

A “co-expressing line” was generated by driving the expression of *UAS-Or42a* with the mutant *(42a4)-Gal4* promoter, creating larvae that expressed *Or42a* in ∼6–7 additional non-endogenous ORNs and ∼3 GRNs. The *(42a4)-Gal4* flies were used as an appropriate “control line” to rule out any effect of the transcription and translation of yeast Gal4 protein in the additional chemosensory neurons. Fifty embryos of a given genotype were placed on a limiting food source (100 mg) as done previously [27]. We compared the survival of both lines on the limited quantities of food, and found no significant differences (Figure 2A). These results indicate that the *Or42a* co-expressing larvae do not have a general decrease in survival when a single limiting food source is present.

It has been shown that larvae need their olfactory system to find secondary food sources [27]. In order to test if addition of another food source could improve survivorship, a second food source was introduced inside the assay chamber at a short distance at 3 days after egg laying as done previously (Figure S1A) [27]. Interestingly, the receptor “co-expressing” larvae showed a significantly lower survival at the end of the assay than the “control” larvae (Figure 2B). These results suggested that the “co-expressing” larvae were unable to exploit the secondary food source placed at a distance as effectively as control, presumably due to deficits in navigating using olfactory cues (Figure 2C). These results indicate that under conditions of limited resources where exploitation of secondary food sources are required, larvae that co-express odor receptors may be at a disadvantage for survival.

In order to directly test performance in a competitive survival assay [27], equal numbers of both “co-expressing” and “control” larvae were added to the arena and required to compete for a limiting primary and secondary food source (Figure S1B). The “co-expressing” larvae showed a significantly lower survival rate compared to the “control” larvae (Figure 2D). These results indicate that the “co-expressing” larvae with disruption of the restricted *Or* expression pattern had a significant disadvantage as compared to the control in a competitive environment.

**Figure 3:**
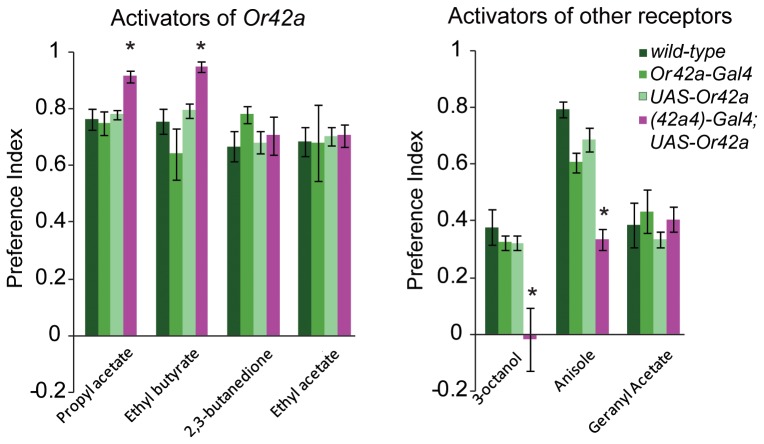
Behavioral responses to some attractive odors is different in larvae co-expressing ***Or42a*** **in multiple neurons.** The mean preference index (PI) of larvae towards attractive odor (10^−2^) is depicted. N = 8 trials, ∼50 larvae/trial, error bars = s.e.m., T-test, **P<0.01.

The comparatively lower survivorship of the “co-expressing” larvae does not occur when embryos are placed directly in a single food source (Figure 2A), but only when they are required to use the olfactory system to find a second food source placed at a distance (Figure 2B,C,D). In order to test whether there are any underlying deficits in olfactory function, we performed a well-established behavior assay towards a point source of odorant [20], [25]. The *Or42a* “co-expressing” larvae showed no major defects in levels of attraction towards odorants normally activating Or42a, and in some instances showed a small but significant increase (Figure 3). Surprisingly, the “co-expressing” larvae did show a dramatic reduction in the behavioral attraction towards two of the three tested odorants that activate other receptors as compared to controls (Figure 3). This effect was odor-specific since attraction towards geranyl acetate, an odor detected by other receptors, was unaffected (Figure 3), perhaps because Or42a is not ectopically co-expressed in all ORNs. It is also possible that the misexpression of *Or42a* in the three neurons innervating the terminal taste organ may also contribute to lower survivorship. While we have not ruled this possibility out, it is unlikely that this is due to odorant-mediated Or42a activity in these gustatory neurons given that the obligate co-receptor, *Orco*, is not expressed in these cells [28].

**Figure 4:**
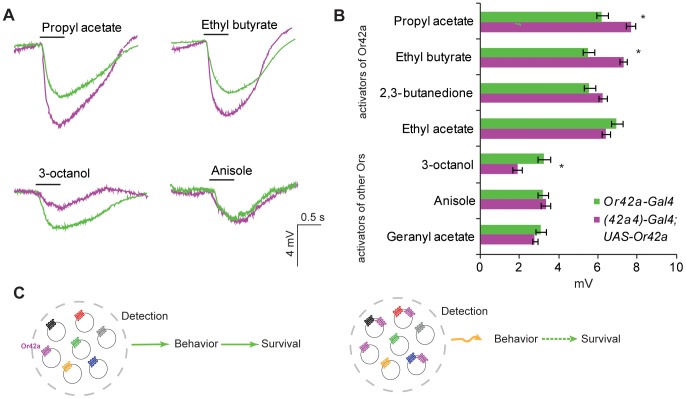
Electrodomogram (EDG) recordings from the larval dome sensillum of larvae co-expressing ***Or42a*** **in multiple nuerons.** Mean EDG response (**A**) trace and (**B**) response to 0.5 sec pulse of indicated odorants (10^−2^). N = 6–8, error bars = s.e.m, T-test, **P<0.01.

The deficit in odor-mediated behavior could be a result of aberrant processing of information in the higher brain centers of the “co-expressing” larvae, or by defects in detection of non-Or42a odors in the periphery, or both. In order to test whether differences in behavioral response in the “co-expressing” larvae were due to differences in odor sensing in the periphery, we performed quantitative electrophysiological analysis. We developed an extracellular recording method, similar to Electroantennograms (EAG), for the larvae where a glass electrode was inserted in the dome sensillum and a reference electrode inserted through the body wall. The receptor potentials were recorded in response to odor stimuli and quantified in the Electrodomograms (EDG). The EDG responses to two odorants detected by Or42a (propyl acetate and ethyl butyrate) were significantly higher in the “co-expressing” larvae (Figure 4A, B). This is expected given that Or42a is now expressed in more neurons, and also consistent with the increase in behavioral attraction towards them (Figure 3). However, the response to a non-Or42a odorant, 3-octanol, was significantly decreased in the “co-expressing” larval dome sensillum (Figure 4A,B) suggesting that a reduction in sensitivity was responsible for the lower behavioral attraction (Figure 3). The responses to two other odorants were unaffected (Figure 4B). Interestingly, the behavioral reduction to anisole is not due to an electrophysiological defect in the periphery suggesting the possibility of central mechanisms participating in the behavioral changes as well. Taken together these EDG experiments demonstrate that the “co-expressing” larvae showed differences in peripheral sensitivity to odorants as compared to control larvae, which could underlie changes in olfactory behavior and overall survival when navigating towards a secondary food source is required.

## Discussion

Our results suggest that co-expression of odor receptor *Or42a* may lead to a decrease in sensitivity and behavior towards odorants detected by the endogenous odor receptors (Figure 4C). Although beyond the scope of this analysis, there could be various reasons for this deficit. Several possibilities exist even at the peripheral level for the incompatibility: levels of endogenous receptor protein present on the dendritic membrane surface may be lower, or essential signal transduction partners such as Orco may be titrated away by the co-expressed receptor. Alternatively, some odors while activating the endogenous receptor may simultaneously inhibit the co-expressed receptor in the same cell leading to a reduction in activity. Moreover it is not known whether the formation of novel heteromeric combinations of receptors can lead to inefficient receptors.

The coding of odor mixtures as present in a complex food source such as yeast may also present additional challenges to an olfactory system that co-expresses multiple receptors. It has recently been shown that activation of one neuron can inhibit the activities of other adjoining neurons sharing the same electrically isolated compartment, such as a sensillum, through non-synaptic mechanisms [29]. These effects could be pronounced in the larval dome sensillum, which contains 21 ORNs that are present in close vicinity, particularly if several neurons were activated by similar odorants due to misexpression in the “co-expressing” larvae.

Finally, the competitive survival analyses provides the first evidence that breakdown of the one-receptor-per-neuron rule in the olfactory system imparts a significant disadvantage in an environment where food is limiting and organisms are required to utilize their olfactory system to navigate towards additional food sources.

## Materials and Methods

### Behavioral Tests

Larval behavior assays were performed as described in [25], [30], with a minor modification that odors were presented in caps of 0.2 ml PCR tubes instead of filter paper discs. Two 0.2 ml PCR flat caps to which 25 µl of odor at 10^−2^ concentration in paraffin oil was added, were placed on opposite sides on a thin layer of 1% agarose in a 100 mm×15 mm Petri (Fisher) dish. Approximately 50–70 early third instar larvae (wandering third instars that climbed out of food were excluded) were placed in the center of the dish and allowed to navigate to odors for 5 min, after which a preference index (PI) was calculated as described in [25]. The number of larvae, O, on the odor half of the Petri dish, and, C, on the control half; PI = (O−C)/(O+C). The PI ranges from 1, complete attraction to 0, neutral, and −1 complete avoidance.

### Survival and Competition Assays

Survival and competition assays were performed following the protocol described in [27]. Genotypes of eclosed flies from competition assays were determined using PCR primers that selectively amplify *UAS-Or42a* transgene. Positive control primers for gDNA isolation were for synaptotagmin.

Embryo collection: Adult flies 4–7 days old were placed onto an egg laying cage with a 60 mm×15 mm (Falcon) grape juice agar plate containing yeast. Cages were placed in the dark at 25 degree Celsius, 70–80% humidity overnight 0 days after egg laying (AEL).

Survival assay: At ∼14–16 hours AEL embryos were collected and introduced into the behavior assay plate a 150 mm×15 mm Petri dish (Falcon) with a thin layer of 2.5% agarose. The primary food source was 100 mg of cornmeal, molasses, and yeast medium which was dispensed into a 9 mm plastic tap (USA Scientific). The secondary food source was identical to the primary with the addition of 70 mg of live yeast paste. All experiments were carried out in the dark at 25 degree Celsius and 70–80% relative humidity.

Survival assay supplemented with secondary food source: Primary food source was placed in the center of the behavior assay plate and 50 embryos were placed on the food. At 3 days after egg laying (AEL) while larvae were consuming the primary food source, the secondary food source was placed 70 mm from center of the assay plate. Control plates received an empty cap. Starting at 10 days AEL, newly emerged flies were collected and counted daily until 20 days AEL. To measure distribution of larvae at 5 days AEL, additional survival assay experiments were run in parallel and on 5 days AEL, the number of larvae in either the primary or secondary food source was counted.

Survival competition assay: The competition assay was carried out exactly as the survival, except 50 embryos from each of the two genotypes were introduced into the primary food source at the same time. Genotypes of emerging flies were determined by PCR analysis of individual flies. Genomic DNA was extracted by crushing individual flies in buffer (10 mM Tris-HCL, 5 mM EDTA, 25 mM NaCl, 200 µg/ml proteinase K). The following primers were used:

Synaptotagmin (positive control)

Primer 1: CGGATCCCTATGTCAAGGTG

Primer 2: TCTGGTCGTGCTTCGAGAAG

Or(42a4)-G4; UAS-Or42a

Primer 1(UAS primer): GCAACTACTGAAATCTGCCAAG

Primer 2 (Or42a primer): AATAACAGGACGCAGGCAGT

Cumulative eclosion rates for the survival and competition assay were calculated as the number of adults emerged by “N” days AEL divided by the total number of embryos introduced.

### Electrophysiology

Electrodomogram (EDG) recordings were made from the dome sensillum of third instar larvae by immobilizing the larvae onto a toothpick using strips of parafilm tightly wrapped around the body, such that only the dorsal organ including the dome sensillum was exposed. A reference electrode was inserted through the parafilm into the larval body and a glass-recording electrode containing sensillar lymph ringer (18) was inserted in to the dome sensilla. The EDG signals were amplified using a WPI amplifier (Gain 100, High pass 1.0 Hz, Low pass 0.1 Hz), and Digidata 1440A. Measured pulse of odor stimulus was applied using a Syntech CS-55 system where a 0.5 sec puff of filtered air was blown through an odor delivery cartridge (as in [31], [32]) containing a filter paper with 50 µl of 10^−2^ odorant dissolved in paraffin oil, chemical were of the highest purity available from Sigma Aldrich (>98%). Mean EDG traces were generated in Clampfit 10.3 (pCLAMP 10, Molecular Devices) by overlaying individual responses using the average trace module.

### Staining

The brains of *Or42a-Gal4/UAS-mcd8:GFP;UAS-mcd8:GFP/+* and *Or(42a4)-Gal4/UAS-mcd8:GFP;UAS-mcd8:GFP/+* larvae were dissected and stained using primary antibodies mouse anti-nc82 1:10 and rabbit anti-GPP 1∶1000 (DSHB) and secondary antibodies Alexa 488 goat anti-rabbit 1∶200 and Alexa 546 goat anti-mouse 1∶200 (Invitrogen) as described in [20]. Fluorescence was visualized using a Leica SP5 inverted confocal microscope using 23× and 40× objectives.

### Genetics

Fly stocks were maintained on standard cornmeal, yeast, and molasses medium at 25°C. Wild-type (wCS) stock is *w^1118^* backcrossed 5 generations to Canton S. The *Or42a-Gal4* and the *(42a4)-Gal4* promoter transgenic constructs and flies are described in [6]. *UAS-Or42a* flies were a kind gift of J. R. Carlson.

## Supporting Information

Figure S1 (**A**) Schematic of survival assay. At day 1, fifty embryos of a particular genotype are placed on the primary food source in the center. At day 3 a second food source is introduced on the same plate. Starting from 10 days after egg laying pupae are represented as yellow ovals and empty pupal cases as blank ovals. (**B**) Schematic of competition assay where 50 larvae of each of the two genotypes are added together to the primary food source.
(TIF)

Video S1 **Animation of confocal Z-stacks of the larval head showing GFP+ neurons innervating the dome sensillum and the terminal organ in larvae expressing** ***UAS-mcd8:GFP*** **under the control of the wild-type** ***Or42a-Gal4*** **promoter.**
(AVI)

Video S2 **Animation of confocal Z-stacks of the larval head showing GFP+ neurons innervating the dome sensillum and the terminal organ in larvae expressing** ***UAS-mcd8:GFP*** **under the control of the mutant** ***(42a4)-Gal4*** **promoter.**
(AVI)

Video S3 **Animation of confocal Z-stacks of the larval central nervous system showing GFP+ neurons innervating the larval antennal lobe and subesophageal ganglion in larvae expressing** ***UAS-mcd8:GFP*** **under the control of the wild-type** ***Or42a-Gal4*** **promoter.**
(AVI)

Video S4 **Animation of confocal Z-stacks of the larval central nervous system showing GFP+ neurons innervating the larval antennal lobe and subesophageal ganglion in larvae expressing** ***UAS-mcd8:GFP*** **under the control of the mutant** ***(42a4)-Gal4*** **promoter.**
(AVI)

## References

[1] Fuss SH, Omura M, Mombaerts P (2007) Local and cis effects of the H element on expression of odorant receptor genes in mouse. Cell 130: 373–384. 17662950 10.1016/j.cell.2007.06.023

[2] Shykind BM, Rohani SC, O'Donnell S, Nemes A, Mendelsohn M, et al (2004) Gene switching and the stability of odorant receptor gene choice. Cell 117: 801–815. 15186780 10.1016/j.cell.2004.05.015

[3] Serizawa S, Miyamichi K, Nakatani H, Suzuki M, Saito M, et al (2003) Negative feedback regulation ensures the one receptor-one olfactory neuron rule in mouse. Science 302: 2088–2094. 14593185 10.1126/science.1089122

[4] Fuss SH, Ray A (2009) Mechanisms of odorant receptor gene choice in Drosophila and vertebrates. Mol Cell Neurosci 41: 101–112. 19303443 10.1016/j.mcn.2009.02.014

[5] Tichy AL, Ray A, Carlson JR (2008) A new Drosophila POU gene, pdm3, acts in odor receptor expression and axon targeting of olfactory neurons. J Neurosci 28: 7121–7129. 18614681 10.1523/JNEUROSCI.2063-08.2008 PMC2572001

[6] Ray A, van der Goes van Naters W, Carlson JR (2008) A Regulatory Code for Neuron-Specific Odor Receptor Expression. PLoS Biol 6: e125. 18846726 10.1371/journal.pbio.0060125 PMC2430909

[7] Ray A, van der Goes van Naters W, Shiraiwa T, Carlson JR (2007) Mechanisms of odor receptor gene choice in Drosophila. Neuron 53: 353–369. 17270733 10.1016/j.neuron.2006.12.010 PMC1986798

[8] Bai L, Goldman AL, Carlson JR (2009) Positive and negative regulation of odor receptor gene choice in Drosophila by acj6. J Neurosci 29: 12940–12947. 19828808 10.1523/JNEUROSCI.3525-09.2009 PMC2782464

[9] Tom W, de Bruyne M, Haehnel M, Carlson JR, Ray A (2011) Disruption of olfactory receptor neuron patterning in Scutoid mutant Drosophila. Mol Cell Neurosci 46: 252–261. 20875862 10.1016/j.mcn.2010.09.008 PMC3019251

[10] Jafari S, Alkhori L, Schleiffer A, Brochtrup A, Hummel T, et al (2012) Combinatorial activation and repression by seven transcription factors specify Drosophila odorant receptor expression. PLoS Biol 10: e1001280. 22427741 10.1371/journal.pbio.1001280 PMC3302810

[11] Magklara A, Yen A, Colquitt BM, Clowney EJ, Allen W, et al (2011) An epigenetic signature for monoallelic olfactory receptor expression. Cell 145: 555–570. 21529909 10.1016/j.cell.2011.03.040 PMC3094500

[12] Sim CK, Perry S, Tharadra SK, Lipsick JS, Ray A (2012) Epigenetic regulation of olfactory receptor gene expression by the Myb-MuvB/dREAM complex. Genes Dev 26: 2483–2498. 23105004 10.1101/gad.201665.112 PMC3505819

[13] Clowney EJ, LeGros MA, Mosley CP, Clowney FG, Markenskoff-Papadimitriou EC, et al (2012) Nuclear aggregation of olfactory receptor genes governs their monogenic expression. Cell 151: 724–737. 23141535 10.1016/j.cell.2012.09.043 PMC3659163

[14] Mombaerts P (2004) Odorant receptor gene choice in olfactory sensory neurons: the one receptor-one neuron hypothesis revisited. Current Opinion in Neurobiology 14: 31–36. 15018935 10.1016/j.conb.2004.01.014

[15] Serizawa S, Miyamichi K, Sakano H (2005) Negative feedback regulation ensures the one neuron-one receptor rule in the mouse olfactory system. Chem Senses 30 Suppl 1i99–i100. 15738216 10.1093/chemse/bjh133

[16] Lewcock JW, Reed RR (2004) A feedback mechanism regulates monoallelic odorant receptor expression. Proceedings of the National Academy of Sciences of the United States of America 101: 1069–1074. 14732684 10.1073/pnas.0307986100 PMC327152

[17] Goldman AL, Van der Goes van Naters W, Lessing D, Warr CG, Carlson JR (2005) Coexpression of two functional odor receptors in one neuron. Neuron 45: 661–666. 15748842 10.1016/j.neuron.2005.01.025

[18] Fishilevich E, Vosshall LB (2005) Genetic and functional subdivision of the Drosophila antennal lobe. Curr Biol 15: 1548–1553. 16139209 10.1016/j.cub.2005.07.066

[19] Stortkuhl KF, Kettler R, Fischer S, Hovemann BT (2005) An increased receptive field of olfactory receptor Or43a in the antennal lobe of Drosophila reduces benzaldehyde-driven avoidance behavior. Chem Senses 30: 81–87. 15647466 10.1093/chemse/bji003

[20] Kreher SA, Kwon JY, Carlson JR (2005) The molecular basis of odor coding in the Drosophila larva. Neuron 46: 445–456. 15882644 10.1016/j.neuron.2005.04.007

[21] Fishilevich E, Domingos AI, Asahina K, Naef F, Vosshall LB, et al (2005) Chemotaxis behavior mediated by single larval olfactory neurons in Drosophila. Curr Biol 15: 2086–2096. 16332533 10.1016/j.cub.2005.11.016

[22] Ramaekers A, Magnenat E, Marin EC, Gendre N, Jefferis GS, et al (2005) Glomerular maps without cellular redundancy at successive levels of the Drosophila larval olfactory circuit. Curr Biol 15: 982–992. 15936268 10.1016/j.cub.2005.04.032

[23] Gerber B, Stocker RF (2007) The Drosophila larva as a model for studying chemosensation and chemosensory learning: a review. Chem Senses 32: 65–89. 17071942 10.1093/chemse/bjl030

[24] Vosshall LB, Stocker RF (2007) Molecular architecture of smell and taste in Drosophila. Annu Rev Neurosci 30: 505–533. 17506643 10.1146/annurev.neuro.30.051606.094306

[25] Kreher SA, Mathew D, Kim J, Carlson JR (2008) Translation of sensory input into behavioral output via an olfactory system. Neuron 59: 110–124. 18614033 10.1016/j.neuron.2008.06.010 PMC2496968

[26] Python F, Stocker RF (2002) Adult-like complexity of the larval antennal lobe of D. melanogaster despite markedly low numbers of odorant receptor neurons. J Comp Neurol 445: 374–387. 11920714 10.1002/cne.10188

[27] Asahina K, Pavlenkovich V, Vosshall LB (2008) The survival advantage of olfaction in a competitive environment. Curr Biol 18: 1153–1155. 18674910 10.1016/j.cub.2008.06.075 PMC2575080

[28] Kwon JY, Dahanukar A, Weiss LA, Carlson JR (2011) Molecular and cellular organization of the taste system in the Drosophila larva. J Neurosci 31: 15300–15309. 22031876 10.1523/JNEUROSCI.3363-11.2011 PMC3225198

[29] Su CY, Menuz K, Reisert J, Carlson JR (2012) Non-synaptic inhibition between grouped neurons in an olfactory circuit. Nature 492: 66–71. 23172146 10.1038/nature11712 PMC3518700

[30] Aceves-Pina EO, Quinn WG (1979) Learning in normal and mutant Drosophila larvae. Science 206: 93–96. 17812455 10.1126/science.206.4414.93

[31] Turner SL, Ray A (2009) Modification of CO2 avoidance behaviour in Drosophila by inhibitory odorants. Nature 461: 277–281. 19710651 10.1038/nature08295

[32] Turner SL, Li N, Guda T, Githure J, Carde RT, et al (2011) Ultra-prolonged activation of CO2-sensing neurons disorients mosquitoes. Nature 474: 87–91. 21637258 10.1038/nature10081 PMC3150595

