# Supplementary figures and images for: Advantage of the Highly Restricted Odorant Receptor Expression Pattern in Chemosensory Neurons of Drosophila
Source: PLoS One. 2013 Jun 19;8(6):e66173. doi: 10.1371/journal.pone.0066173 (PMC3686798; doi:10.1371/journal.pone.0066173)

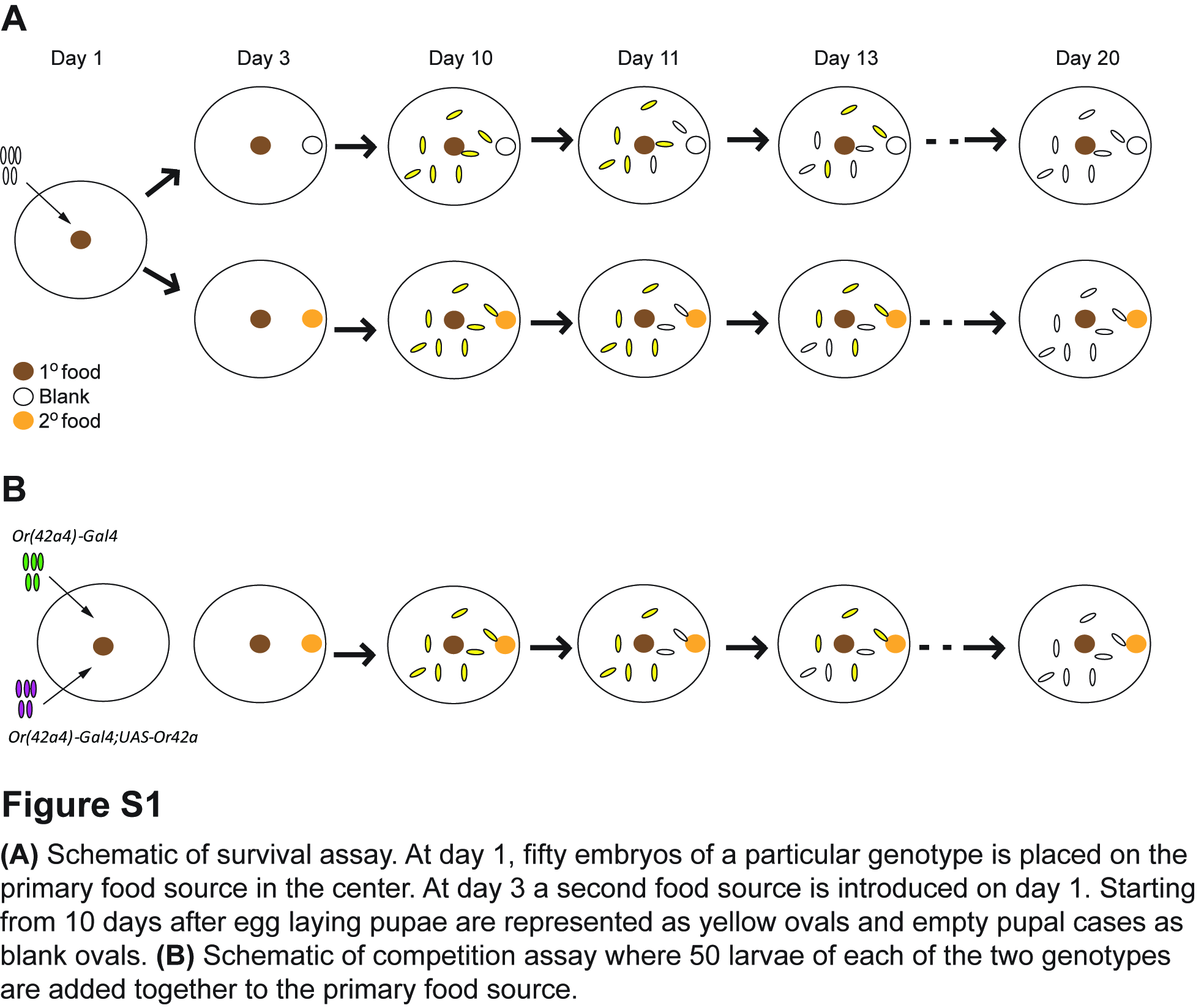

Supplement: Figure S1 — (A) Schematic of survival assay. At day 1, fifty embryos of a particular genotype are placed on the primary food source in the center. At day 3 a second food source is introduced on the same plate. Starting from 10 days after egg laying pupae are represented as yellow ovals and empty pupal cases as blank ovals. (B) Schematic of competition assay where 50 larvae of each of the two genotypes are added together to the primary food source. (TIF) [file pone.0066173.s001.tif]
